# Draft genome of the lowland anoa (*Bubalus depressicornis*) and comparison with buffalo genome assemblies (Bovidae, Bubalina)

**DOI:** 10.1093/g3journal/jkac234

**Published:** 2022-09-16

**Authors:** Stefano Porrelli, Michèle Gerbault-Seureau, Roberto Rozzi, Rayan Chikhi, Manon Curaudeau, Anne Ropiquet, Alexandre Hassanin

**Affiliations:** Department of Natural Sciences, Faculty of Science and Technology, Middlesex University, London NW4 4BT, UK; Institut Systématique Evolution Biodiversité (ISYEB), Sorbonne Université, MNHN, CNRS, EPHE, UA, 75005 Paris, France; Museum für Naturkunde, Leibniz-Institut für Evolutions- und Biodiversitätsforschung, 10115 Berlin, Germany; German Centre for Integrative Biodiversity Research (iDiv) Halle-Jena-Leipzig, 04103 Leipzig, Germany; Institut Pasteur, Université Paris Cité, Sequence Bioinformatics, 75015 Paris, France; Institut Systématique Evolution Biodiversité (ISYEB), Sorbonne Université, MNHN, CNRS, EPHE, UA, 75005 Paris, France; Department of Natural Sciences, Faculty of Science and Technology, Middlesex University, London NW4 4BT, UK; Institut Systématique Evolution Biodiversité (ISYEB), Sorbonne Université, MNHN, CNRS, EPHE, UA, 75005 Paris, France

**Keywords:** Bovidae, *Bubalus depressicornis*, lowland anoa, genome assembly, de novo assembly

## Abstract

Genomic data for wild species of the genus *Bubalus* (Asian buffaloes) are still lacking while several whole genomes are currently available for domestic water buffaloes. To address this, we sequenced the genome of a wild endangered dwarf buffalo, the lowland anoa (*Bubalus depressicornis*), produced a draft genome assembly and made comparison to published buffalo genomes. The lowland anoa genome assembly was 2.56 Gbp long and contained 103,135 contigs, the longest contig being 337.39 kbp long. N50 and L50 values were 38.73 and 19.83 kbp, respectively, mean coverage was 44× and GC content was 41.74%. Two strategies were adopted to evaluate genome completeness: (1) determination of genomic features with de novo and homology-based predictions using annotations of chromosome-level genome assembly of the river buffalo and (2) employment of benchmarking against universal single-copy orthologs (BUSCO). Homology-based predictions identified 94.51% complete and 3.65% partial genomic features. De novo gene predictions identified 32,393 genes, representing 97.14% of the reference’s annotated genes, whilst BUSCO search against the mammalian orthologs database identified 71.1% complete, 11.7% fragmented, and 17.2% missing orthologs, indicating a good level of completeness for downstream analyses. Repeat analyses indicated that the lowland anoa genome contains 42.12% of repetitive regions. The genome assembly of the lowland anoa is expected to contribute to comparative genome analyses among bovid species.

## Introduction

The lowland anoa, *Bubalus depressicornis* (Smith 1827), is a wild dwarf buffalo endemic to Sulawesi and Buton Islands, where it can be found in sympatry with the mountain anoa, *Bubalus quarlesi* (Ouwens 1910). Both anoa species are currently classified as endangered with declining populations due to hunting and habitat loss (Burton *et al.* 2016). Because of their singular appearance, they were initially described in their own genus *Anoa* (Ouwens 1910). However, *Anoa* was not regarded as a valid genus in more recent classifications, in which both anoa species were ascribed to the genus *Bubalus*, together with the wild water buffalo—*Bubalus arnee* (Kerr 1792) and the tamaraw—*Bubalus mindorensis* (Heude 1888; Groves 1969; IUCN 2022). Molecular studies based on mitochondrial sequences have supported a sister-group relationship between *B. depressicornis* and *B. quarlesi* (Schreiber *et al.* 1999; Priyono *et al.* 2020). In addition, the mitogenome of the lowland anoa was found to be equally distant from those of the 2 types of domestic water buffalo, the river buffalo from the Indian subcontinent and Mediterranean countries and the swamp buffalo from China and Southeast Asia (Hassanin *et al.* 2012). Since the same phylogenetic pattern was recovered from the analyses of 2 nuclear datasets, one based on 30 autosomal genes and the other based on 2 genes of the Y chromosome, Curaudeau *et al.* (2021) have concluded the existence of 2 species of domestic buffaloes: *Bubalus bubalis* (Linnaeus 1758) for the river buffalo and *Bubalus kerabau* (Fitzinger 1860) for the swamp buffalo, which diverged during the Pleistocene at around 0.84 Mya. As discussed in Curaudeau *et al.* (2021), the 2 domestic species can easily be distinguished based on coat and horn characteristics (Castelló 2016), and they have different karyotypes: *B. bubalis* has 2*n* = 50 chromosomes with a fundamental number (FN) equal to 58; whereas *B. kerabau* has 2*n* = 48 chromosomes and FN = 56 (Nguyen *et al.* 2008).

With rapid progress and cost reduction in sequencing technologies, many whole genomes of domestic bovid species have been sequenced. Whole-genome sequencing has allowed the identification of variants involved in domestication and genetic improvement for several livestock species such as cattle and buffaloes (Zimin *et al.* 2009; Canavez *et al.* 2012; Li *et al.* 2020; Rosen *et al.* 2020). Chromosome-level genome assemblies include those of the domestic cow, *Bos taurus* (Zimin *et al.* 2009), the domestic river buffalo, *B. bubalis* (Deng *et al.* 2016), the swamp buffalo, *B. kerabau* [reported as *Bubalus carabanensis* in Luo *et al.* (2020) but see Curaudeau *et al.* (2021) for further taxonomic information], the domestic Yak, *Bos grunniens* (Zhang *et al.* 2021) and the zebu cattle, *Bos indicus* (Canavez *et al.* 2012). Whereas a total of 8 chromosome- and scaffold-level genome assemblies are publicly available for domestic buffaloes, there are currently no genome data available for wild species of the genus *Bubalus*. To fill this gap, a biopsy of a living lowland anoa was used for next-generation sequencing, and a draft genome was assembled de novo for comparison to other buffalo genome assemblies available in international databases such as NCBI (National Center for Biotechnology Information) and BIG_GWH (Beijing Institute of Genomics Genome Warehouse database).

## Materials and methods

### DNA extraction, library preparation, and genome sequencing

**Fig. 1.**
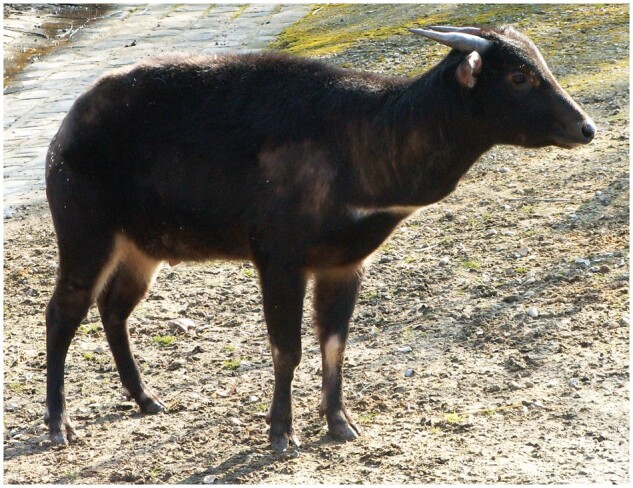
Lowland anoa (*Bubalus depressicornis*) housed at the *Ménagerie du Jardin des Plantes* (© Alexandre Hassanin—MNHN).

A living male adult of lowland anoa, named Yannick, was sampled at the *Ménagerie du Jardin des Plantes* of the Muséum national d’Histoire naturelle (MNHN, Paris, France; Fig. 1). A skin biopsy was performed in 2006 by a veterinary surgeon following protocols approved by the MNHN and in line with ethical guidelines. The same biopsy was previously used to determine its karyotype (2*n* = 48; FN = 58; Nguyen *et al.* 2008). DNA was extracted using the DNeasy Blood and Tissue Kit (Qiagen, Hilden, Germany) following the manufacturer’s protocol. DNA quantification was performed with a Qubit 2.0 Fluorometer with Qubit dsDNA HS Assay Kit (Thermo Fischer Scientific, Walthan, MA, USA). Library preparation and sequencing were conducted at the *Institut du Cerveau et de la Moelle épinière*. The sample was sequenced on a NextSeq 500 Illumina system generating 2 × 151 bp reads using the NextSeq 500 High Output Kit v2 with 300 cycles and aiming for an insert size of 350 bp.

### De novo assembly

Data quality was assessed with FastQC v.0.11.5 (https://www.bioinformatics.babrah am.ac.uk/projects/fastqc/) and results were collated with MultiQC v1.12 (Ewels *et al.* 2016). Raw reads were quality-trimmed and adapter sequences and contaminants removed with Trimmomatic v.0.36 (Bolger *et al.* 2014) with the following parameters: “ILLUMINACLIP: TruSeq3 -PE.fa:2:30:10 LEADING:33 TRAILING:3 SLIDINGWINDOW:4:15 MINLEN:36.” Data quality of quality-trimmed reads was reassessed with FastQC. A de novo assembly was performed with MaSuRCA v.3.3.1 (Zimin *et al.* 2013, 2017) using recommended parameters for mammalian genomes and paired-end Illumina-only data, as indicated in Zimin *et al.* (2017). The mean and standard deviation for the Insert size were estimated with an “estimate-insert-size” script (https://gist.github.com/rchikhi/7281991). Paired-end reads were error corrected using QuorUM (Marçais *et al.* 2015) and assembled into super-reads using a k-mer size of 99, as selected by the MaSuRCA assembler. The super-reads were then assembled into contigs using the CABOG assembler, part of the MaSuRCA pipeline (Zimin *et al.* 2017), followed by gap closing with the paired-end information (Zimin *et al.* 2013).

### Assembly quality assessment

Genome assemblies publicly available for *Bubalus* and *Syncerus* genera were retrieved from NCBI and BIG_GWH for quality comparison and assessment. The dataset included 2 assemblies at the chromosome level for the river buffalo (*B. bubalis*) with a coverage of 100× and 572×, 4 scaffold-level draft assemblies of river buffalo with coverage ranging between 69× and 119×, one chromosome-level assembly of swamp buffalo (*B. kerabau*) with a mean coverage of 65×, and one scaffold-level draft assembly of the African buffalo (*Syncerus caffer*) with 162× coverage. The 8 retrieved assemblies were sequenced and assembled with different methods, summarized in Table 1.

**Table 1.** Information regarding genome assemblies available for buffalo species.

Species/assembly name	Breed	Geographic location	ID	Assembly accession no	Sequencing technology	Assembly method	Coverage	Assembly level
*Bubalus bubalis* NDDB_SH_1_ (RefSeq)	Murrah	India	NDDB_SH_1	GCF_019923935.1	PacBio Sequel; 10X and BioNano Optical Map	Falcon+Scaff10X+BioNano v. 2019-02-25	572×	Chromosome
*Bubalus bubalis* Jaffrabadi_v3.0	Jaffrabadi	India	AAUIN_1	GCA_000180995.3	454; Illumina NextSeq 500	MaSuRCA v. 2.3.2b	100×	Scaffold
*Bubalus bubalis* UOA_WB_1	Mediterranean	Italy	UOA_WB_1	GCA_003121395.1	PacBio	Falcon-Unzip v. 1.8.7	69×	Chromosome
*Bubalus bubalis* Bubbub1.0	Bangladesh	Bangladesh	Bubbub1.0	GCA_004794615.1	Illumina HiSeq 2000	Soapdenovo v. 2.04	119×	Scaffold
*Bubalus bubalis* ASM299383v1	Egyptian	Egypt	EGYBUF_1.0	GCA_002993835.1	SOLiD	Velvet v. 1.1; Bowtie2 v. 2.1.0; SHRiMP v. 2.2.3	70×	Scaffold
*Bubalus bubalis* UMD_CASPUR_WB_2.0	Mediterranean	United States	UMD_CASPUR_WB_2.0	GCA_000471725.1	Illumina GAIIx; Illumina HiSeq; 454	MaSuRCA v. 1.8.3	70×	Scaffold
Bubalus depressicornis* MNHNYannick_LA_1	—	Indonesia	MNHNYannick_LA_1	Assembled MaSuRCA	Illumina NextSeq 500	MaSuRCA v. 3.3.1	44×	Scaffold
*Bubalus kerabau* CUSA_SWP	Fuzhong	China	CUSA_SWP	GWHAAJZ00000000	PacBio 57.8	Wtdbg 1.2.8	65×	Chromosome
*Syncerus caffer* ASM640878v2	African Buffalo	South Africa	ABF221	GCA_006408785.2	Illumina HiSeq	Platanus v. 1.2.4	162×	Scaffold

* This study.

The quality of the lowland anoa genome assembly was assessed with QUAST-LG v.5.0.1 (Mikheenko *et al.* 2018) using the river buffalo NDDB_SH_1 genome assembly (Deng *et al.* 2016) as a reference. The default parameters for mammalian genomes were used to compare all assemblies in QUAST-LG: “MODE: large, threads: 50, eukaryotic: true, minimum contig length: 3,000, minimum alignment length: 500, ambiguity: 1, threshold for extensive misassembly size: 7,000.” All analyzed assemblies were aligned to the river buffalo NDDB_SH_1 assembly and results were plotted with Circos v. 0.69.8 (Krzywinski *et al.* 2009) and Jupiter consistency plots (Chu 2018).

We adopted 2 different strategies to evaluate genome completeness. Firstly, genomic features were predicted with the homology-based method by aligning the lowland anoa genome to that of the annotated river buffalo reference genome (NDDB_SH_1 and relative annotations retrieved from NCBI). Secondly, we used a de novo gene prediction method with GlimmerHMM v3.0.4 (Majoros *et al.* 2004). Thirdly, we employed benchmarking against universal single-copy orthologs (BUSCO v5.2.2; Manni *et al.* 2021) using the mammalia_odb10 dataset (2021 February 19, number of genomes: 24, number of BUSCOs: 9,226) from OrthoDB (Kriventseva *et al.* 2019) and compared to other buffalo genome assemblies already deposited on NCBI and BIG_GWH (Table 1).

### Repeats and gene annotation

Repetitive regions in the lowland anoa genome were identified, annotated, and masked with RepeatMasker v.4.1.2-p1 (Tarailo-Graovac and Chen 2009). Firstly, a de novo repeat library was constructed from the genome assembly with RepeatModeler v.2.0.2a. RepeatMasker was used with default parameters to produce a homolog-based repeat library and mask the genome’s repetitive regions. The scripts “*calcDivergenceFromAlign.pl”* and “*createRepeatLandscape*.*pl”* were used to calculate the Kimura divergence values and to plot the resulting repeat landscape. The repeat landscape of *B. taurus* was retrieved from the RepeatMasker database for visual comparison.

## Results and discussion

### Whole-genome sequencing and data QC

Whole-genome sequencing generated 991,437,058 paired-end reads with a length of 151 bp. Quality trimming removed 46,616,722 low-quality, adapter-contaminated, and PCR-duplicated reads, representing approximately 0.5% of the total reads. A total of 944,820,336 clean paired-end reads were generated, covering the lowland anoa genome with an estimated 56× depth based on a genome size of 2.56 Gbp. The estimation of insert size using in-house script returned a mean of 377 and a standard deviation of 83.

### De novo assembly quality metrics

**Fig. 2.**
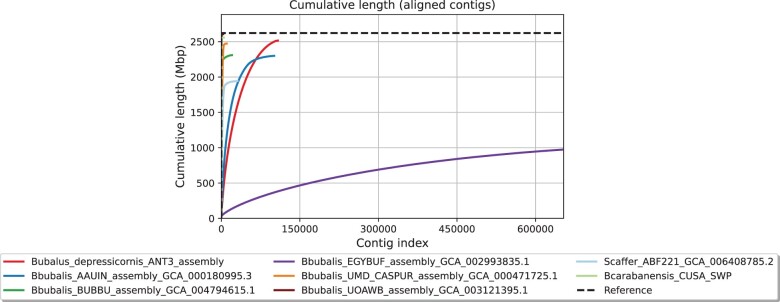
Cumulative length of aligned contigs of the lowland anoa (red line) against the river buffalo NDDB_SH_1 reference genome assembly (dashed line) and compared to other buffalo genome assemblies available on NCBI.

The final lowland anoa genome assembly generated here contained 103,135 contigs, the largest being 337.39 kbp long, an N50 of 38.73 kbp and an L50 of 19.83 kbp (Table 2). The total length was 2.56 Gbp with a mean coverage of 44×, and GC content was 41.74%, in agreement with other published assemblies (between 41.60% and 41.92%, Table 3). When aligned to the NDDB_SH_1 genome assembly, the fraction of the anoa genome assembly was 95.41%, a value comparable to other buffalo genome assemblies (Fig. 2), with a total alignment length of 2,515,453,843 bp. A total of 886 contigs could not be aligned to the river buffalo genome assembly, whilst 8,085 contigs were only partially aligned, resulting in a total unaligned length of 45,224,171 bp, which reflects the discrepancy between the total length of the lowland anoa genome and the total aligned length to the reference river buffalo genome assembly. Partially aligned and unaligned contigs could have resulted from structural variations between the lowland anoa and the reference river buffalo assembly, such as large INDELS (insertion/deletions), as well as repetitive regions and/or alternative haplotypes causing assembly errors. The nature of short-read technology causes difficulties in characterizing genomic regions such as telomeres, centromeres, repetitive, and highly heterochromatic regions (Johnson *et al.* 2005; Low *et al.* 2019; Weissensteiner and Suh 2019), which are notoriously difficult to assemble and could be better resolved with long-read sequencing.

**Table 2.** Draft assembly statistics of the lowland anoa genome.

Contig statistics	value
Total length	2,565,510,706
Number of contigs	103,135
Largest contig	337,395
GC (%)	41.74
N50	38,737
L50	19,832

**Table 3.** Comparison of assembly quality metrics of the lowland anoa (*Bubalus depressicornis*) and other buffalo assemblies.

Name/assembly name (NCBI)	ID	Genome fraction %	Total aligned length	Largest alignment	Scaffolds count	N50	L50	GC%
*Bubalus bubalis* NDDB_SH1 (RefSeq)	NDDB_SH_1	—	—	—	26	116,997,125	9	41.75
*Bubalus bubalis* Jaffrabadi_v3.0	AAUIN_1	83.189	2,299,810,356	834,863	75,621	104,127	9,942	41.78
*Bubalus bubalis* UOA_WB_1	UOA_WB_1	98.851	2,605,694,501	34,949,624	509	117,219,835	9	41.81
*Bubalus bubalis* Bubbub1.0	Bubbub1.0	86.537	2,309,804,413	9,328,338	14,905	7,025,746	116	41.6
*Bubalus bubalis* ASM299383v1	EGYBUF_1.0	36.01	974,053,149	2,013,276	6,313	3,666,815	234	41.92
*Bubalus bubalis* UMD_CASPUR_WB_2.0	UMD_CASPUR_WB_2.0	93.634	2,473,056,510	7,952,377	5,714	1,545,294	508	41.73
*Bubalus depressicornis* MNHNYannick_LA_1	MNHNYannick_LA_1	95.415	2,515,453,834	337,395	103,135	38,737	19,832	41.74
*Bubalus kerabau* CUSA_SWP	CUSA_SWP	97.086	2,557,653,758	23,566,932	1,534	117,253,548	8	41.83
*Syncerus caffer* ASM640878v2	ABF221	73.046	1,942,672,810	4,692,267	13,167	2,448,414	351	41.72

The lowland anoa genome assembly has a modest N50 compared to other buffalo genome assemblies (Table 3), indicating lower levels of contiguity, which is expected due to the short-read output of Illumina sequencing technology (read length = 151 bp). In addition, repeat analysis revealed that 42.12% of the lowland anoa genome is composed of repetitive regions. This, coupled with low-sequence coverage, sequencing and assembly errors, causes breaks in the assembly contiguity (Gnerre *et al.* 2011; Low *et al.* 2019). This is apparent even in high-quality chromosome-level genome assemblies that use multiple sequencing libraries and multiple sequencing technologies, such as the previous human genome assembly GRCh38, which contained hundreds of gaps (International Human Genome Sequencing Consortium 2004). In addition, the chromosome-level genome assemblies retrieved from NCBI (NDDB_SH_1, UOA_WB_1) were sequenced using multiple insert size libraries and sequencing technologies and were intensively verified with multiple methods such as optical mapping, Hi-C, and RH (Deng *et al.* 2016; Low *et al.* 2019).

Moreover, quality metrics of publicly available assemblies are usually limited to reporting N50 and L50 values, which represent the shortest contig length needed to cover 50% of the total assembly size, and the number of contigs whose cumulative length covers 50% of the total assembly size, respectively (Bradnam *et al.* 2013). Such metrics are often used to compare and evaluate performances of the ever-growing assembly and annotation methods and software (Manchanda *et al.* 2020). However, we hereby show that reporting N50 and L50 metrics exclusively can be misleading, as they only provide a standard measure of assembly contiguity whilst omitting information such as gene content and completeness, as well as assembly correctness. Furthermore, N50 values can be artificially raised by deliberately excluding short contigs from analyses and by the presence of undetermined nucleotides (Ns) linking the scaffolded contigs (Gurevich *et al.* 2013). Therefore, to assess the quality of the lowland anoa genome assembly, we generated conventional N50 and L50 metrics and also determined genome completeness in terms of gene content and genome correctness by comparing our assembly to a chromosome-level genome assembly of the river buffalo (*B. bubalis*). In addition, a swamp buffalo (*B. kerabau*, CUSA_SWP) and a more distantly related African buffalo species (*S. caffer*, ABF221) were also included in our comparison.

**Fig. 3.**
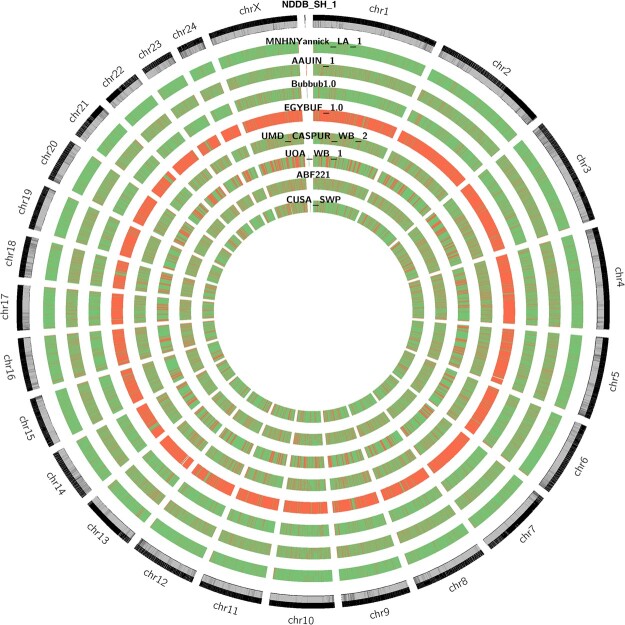
Circos plot of scaffolds mapped to NDD_SH_1 reference genome assembly (*Bubalus bubalis*). Outer circle represents reference sequence with GC% heatmap (0% = white, 69% = black). Inner circles represent assembly tracks, with heatmap representing correct contigs (green) and misassembled blocks (red).

**Fig. 4.**
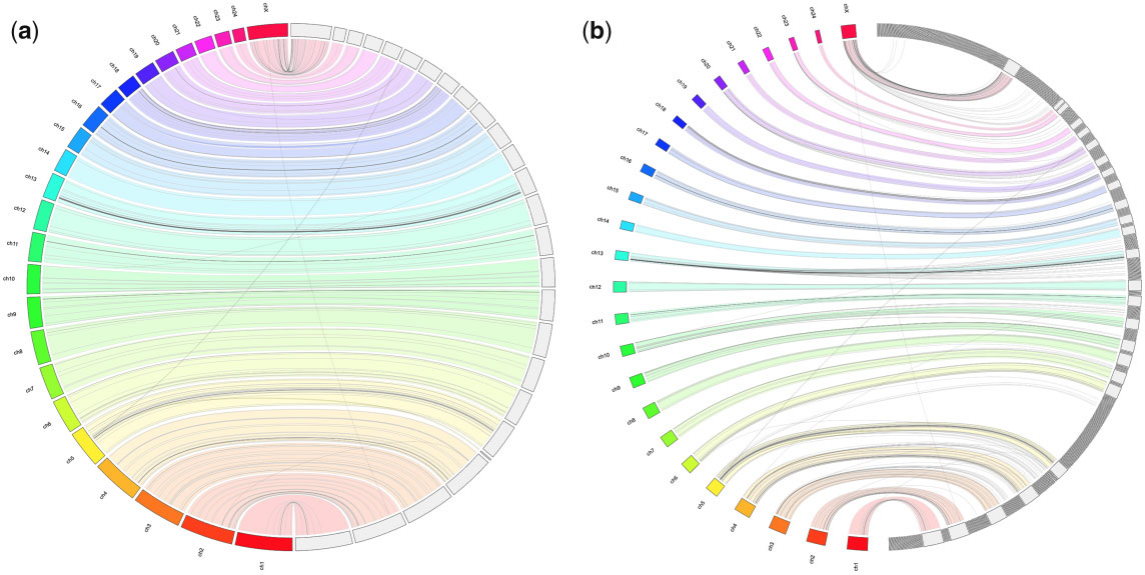
Jupiter consistency plot showing alignment between the river buffalo genome assemblies UO_AWB_1 and NDDB_SH_1. The left of the plots shows the numbered NDDB_SH_1 chromosomes. The right of the plots shows (a) the 26 longest contigs of the UOA_WB_1 assembly needed to cover 100% of the reference genome and (b) all the 509 contigs of the UO_AWB_1 assembly. Colored bands represent synteny between the genomes. Lines represent genomic rearrangements, break points in the scaffolds or assembly errors. The absence of lines connecting the UO_AWB_1 blocks to the NDDB_SH_1 chromosomes indicates contigs that could not be aligned to the reference.

Regardless of the modest N50 value, the lowland anoa genome assembly is in good agreement with the NDDB_SH_1 assembly, with 95.91% of contigs correctly mapped to the 25 reference chromosomes of the river buffalo and fewer misassembled blocks compared to other draft assemblies (Fig. 3). The genome assembly of the Egyptian river buffalo (EGYBUF_1.0) had an abnormally high number of misassembled blocks with respect to the reference genome, followed by the genome assembly of a female Italian river buffalo (UOA_WB_1). To investigate this, misassemblies and structural variation metrics were computed in QUAST-LG (Table 4). The Egyptian river buffalo assembly (EGYBUF_1.0) showed the highest number of mismatches and the highest number of Ns, followed by the Jaffrabadi river buffalo (AAUIN_1). The genome assembly of the African buffalo (*S. caffer*, ABF221) showed a larger number of mismatches (Table 4), but this can be explained by the higher sequence divergence between *Syncerus* and *Bubalus*, as the 2 genera have separated in the Late Miocene (Hassanin *et al.* 2012). Misassemblies and structural variation metrics could not explain the misassembled blocks of the UOA_WB_1 assembly observed in the Circos plot of Fig. 3. However, some of these misassembled blocks could be due to unplaced contigs. To investigate this, the UOA_WB_1 assembly was aligned to the NDDB_SH_1 reference to generate Jupiter consistency plots. When using the largest 26 contigs of the UOA_WB_1 assembly to cover 100% of the reference river buffalo genome, an almost perfect level of synteny was observed (Fig. 4a). Although this result was expected for genomes of the same species, it also indicates a good level of assembly quality in terms of correctness. However, when including all 509 contigs of the UOA_WB_1 assembly, several misassembled regions were observed (Fig. 4b). Three nonexclusive hypotheses can be advanced to interpret this result: possible genomic rearrangements, genome assembly errors, and repetitive regions. Whether the results of the consistency plots are due to the factors mentioned above or other factors, such as contamination, remains speculative. Nevertheless, the results of the quality metric comparison conducted here further indicate the unreliability of using exclusively N50 and L50 metrics when assessing assembly quality. Instead, contiguity metrics should be supplemented with genome completeness and correctness metrics.

**Table 4.** QUAST-LG statistics of all buffalo assemblies with respect to the river buffalo NDDB_SH_1 reference.

	*B. depressicornis* MNHNYannick_LA_1	***B. bubalis*** **AAUIN_1**	*B. bubalis* Bubbub1.0	*B. bubalis* EGYBUF_1.0	*B. bubalis* UMD_CASPUR_WB_2.0	*B. bubalis* UOA_WB_1	***B. kerabau*** **CUSA_SWP**	***S. caffer*** **ABF221**
Misassemblies	4,949	19,238	3,561	131	4,040	1,724	2,111	6,565
Relocations	1,447	13,540	2,761	85	1,434	1,051	1,199	3,397
Translocations	3,203	4,714	757	10	2,569	647	896	3,032
Inversions	299	984	43	36	37	26	16	136
Misassembled contigs	4,550	15,988	1,049	45	1,943	255	533	1,727
Misassembled contigs length	159,179,266	1,334,096,556	2,506,642,146	55,459,162	1,891,377,139	2,639,940,877	2,594,120,526	2,486,555,687
Local misassemblies	7,014	73,267	241,261	6,933	7,100	4,870	9,940	435,454
Possible TEs	164	874	886	10	544	136	158	654
Unaligned mis. contigs	287	2,378	548	2,522	63	104	381	1,324
Unaligned contigs	886 + 8,085 partial	2,555 + 57,865 partial	297 + 7,280 partial	2,806 + 3,472 partial	182 + 3,290 partial	1 + 416 partial	140 + 1110 partial	900 + 7,314 partial
Unaligned length	45,224,171	596,227,806	299,544,303	1,673,093,194	82,826,374	49,291,638	51,316,520	779,611,955
Genome fraction (%)	95.415	83.189	86.537	36.01	93.634	98.851	97.086	73.046
Duplication ratio	1.007	1.425	1.076	1.36	1.034	1.005	1.013	1.045
Mismatches	16,233,421	19,654,061	23,375,163	17,890,296	10,863,130	10,118,782	15,844,866	114,608,168
Indels	1,578,224	746,243	705,955	6,440,610	1,136,878	1,400,310	1,534,735	2,128,964
Indels length	12,654,316	56,163,406	24,209,936	35,356,432	24,745,254	23,411,739	33,123,824	18,236,722
Mismatches per 100 kbp	649	901	1,030	1,895	442	390	622	5,983
Indels per 100 kbp	63	34	31	682	46	54	60	111
Indels (≤5 bp)	1,297,998	598,354	515,830	5,758,980	893,802	1,227,309	1,269,689	1,641,754
Indels (> 5 bp)	280,226	147,889	190,125	681,630	243,076	173,001	265,046	487,210
*N*'s	493,027	850,098,824	138,209,713	328,128,682	73,946,361	373,500	22,116,406	59,283,755
*N*'s per 100 kbp	19.22	22,942	5,040.03	11,097	2,820.18	14.06	840.50	2,131.26

### Genomic features, gene prediction, and annotation

Homology and de novo gene predictions performed on the lowland anoa genome assembly were in agreement with each other and indicated a good level of genome completeness. Results were comparable to other published genome assemblies (Tables 5 and 6), and an improvement over the Bangladeshi river buffalo (Bubbub_1.0), the Egyptian river buffalo (EGYBUF_1.0), and Mediterranean river buffalo (UMD_CASPUR_WB_2.0) assemblies.

**Table 5.** Gene features (CDS and mRNA) predicted with GlimmerHMM.

Name/assembly name (NCBI)	ID	Predicted gene features (unique)	Predicted gene features (≥0 bp)	Predicted gene features (≥300 bp)	Predicted gene features (≥1500 bp)	Predicted gene features (≥3,000 bp)
*Bubalus bubalis* Jaffrabadi_v3.0	AAUIN_1	1,065,654	1,087,174 + 1,214 part	719,235 + 911 part	129,801 + 19 part	24,579 + 7 part
*Bubalus bubalis* UOA_WB_1	UOA_WB_1	1,055,791	1,059,972 + 21 part	762,464 + 17 part	154,594 + 0 part	29,659 + 0 part
*Bubalus bubalis* Bubbub1.0	Bubbub1.0	948,732	958,663 + 101 part	655,839 + 73 part	136,045 + 4 part	27,867 + 1 part
*Bubalus bubalis* ASM299383v1	EGYBUF_1.0	826,048	826,155 + 69 part	530,835 + 37 part	96,365 + 0 part	16,243 + 0 part
*Bubalus bubalis* UMD_CASPUR_WB_2.0	UMD_CASPUR_WB_2.0	963,177	964,473 + 138 part	669,508 + 117 part	134,780 + 5 part	26,448 + 2 part
*Bubalus depressicornis* MNHNYannick_LA_1	MNHNYannick_LA_1	1,027,469	1,023,163 + 5,278 part	702,282 + 4,582 part	131,966 + 204 part	24,994 + 37 part
*Bubalus kerabau* CUSA_SWP	CUSA_SWP	1,042,862	1,046,662 + 87 part	752,170 + 70 part	151,809 + 10 part	29,488 + 6 part
*Syncerus caffer* ASM640878v2	ABF221	1,061,091	1,064,542 + 229 part	750,719 + 171 part	150,033 + 10 part	29,460 + 1 part

**Table 6.** Genes predicted with homology-based prediction method.

Name/assembly name (NCBI)	ID	Genes	Partial genes	Total	**% of reference's annotated genes *(*n = 33,348)**
*Bubalus bubalis* Jaffrabadi_v3.0	AAUIN_1	10,804	20,895	31,699	95.05
*Bubalus bubalis* UOA_WB_1	UOA_WB_1	30,810	1,955	32,765	98.25
*Bubalus bubalis* Bubbub1.0	Bubbub1.0	11,039	20,983	32,022	96.02
*Bubalus bubalis* ASM299383v1	EGYBUF_1.0	1,345	23,770	25,115	75.31
*Bubalus bubalis* UMD_CASPUR_WB_2.0	UMD_CASPUR_WB_2.0	18,656	13,271	31,927	95.74
*Bubalus depressicornis* MNHNYannick_LA_1	MNHNYannick_LA_1	19,148	13,245	32,393	97.14
*Bubalus kerabau* CUSA_SWP	CUSA_SWP	28,349	3,419	31,768	95.26
*Syncerus caffer* ASM640878v2	ABF221	8,763	21,575	30,338	90.97

Interestingly, these 3 assemblies showed higher contiguity (N50) than the draft assembly of the lowland anoa, further indicating the unreliability of using exclusively N50 and L50 metrics when assessing genome assembly quality.

**Fig. 5.**
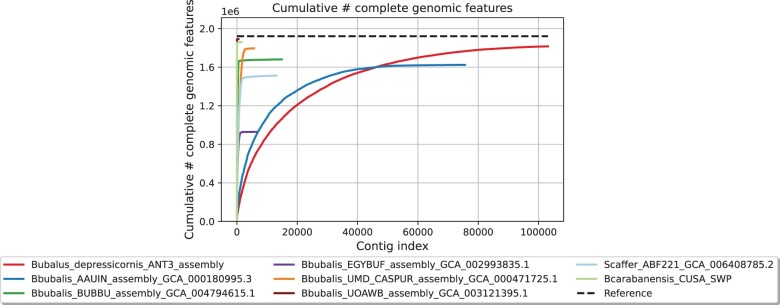
Complete genomic features identified in the lowland anoa assembly and compared to other assemblies using the river buffalo (*Bubalus bubalis*) NDD_SH1 reference sequence and annotations.

Out of the 1,921,249 genomic features annotations of the reference assembly NDDB_SH_1, homology prediction identified 1,815,794 (94.51%) complete and 69,929 (3.63%) partial features in the lowland anoa genome assembly, which is comparable to other published assemblies (Fig. 5), indicating a good level of genome completeness. GlimmerHMM de novo predicted 1,027,469 unique genomic features (mRNA and coding sequences, CDS), which is an improvement over some of the water buffalo assemblies used for quality comparison (Table 5). Homology-based gene prediction identified 32,393 genes in the lowland anoa genome assembly, representing 97.14% of the genes annotated in NDDB_SH_1 (*n* = 33,348). Of these, 59.11% (19,148) were complete and 40.88% (13,245) were partial, probably reflecting the level of fragmentation of the lowland anoa genome assembly. Nevertheless, the total number of genes predicted still represents an improvement over some of the compared assemblies (Table 6).

**Fig. 6.**
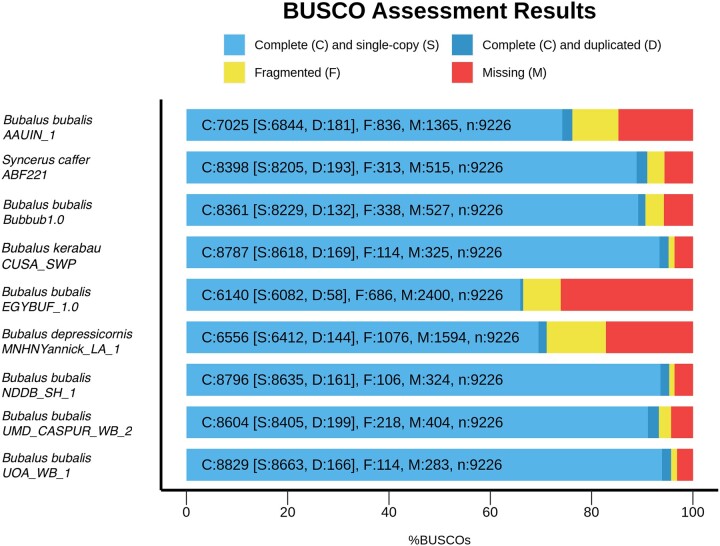
BUSCO results of the genome assembly of the lowland anoa (*Bubalus depressicornis*) compared to other publicly available buffalo genome assemblies.

When predicting mammalian orthologs with BUSCO, the lowland anoa genome assembly contained 6,556 (71.1%) complete BUSCOs, of which 6,412 (69.5%) were single copy and 144 (1.6%) were duplicated. The number of fragmented BUSCOs was 1,076 (11.7%), whilst 1,594 (17.2%) were missing. The BUSCO results indicate an acceptable level of genome completeness (<70%, Simão *et al.* 2015) for downstream analyses for the anoa genome assembly, and a slight improvement over the Egyptian river buffalo assembly (EGYBUF_1.0, Fig. 6).

**Fig. 7.**
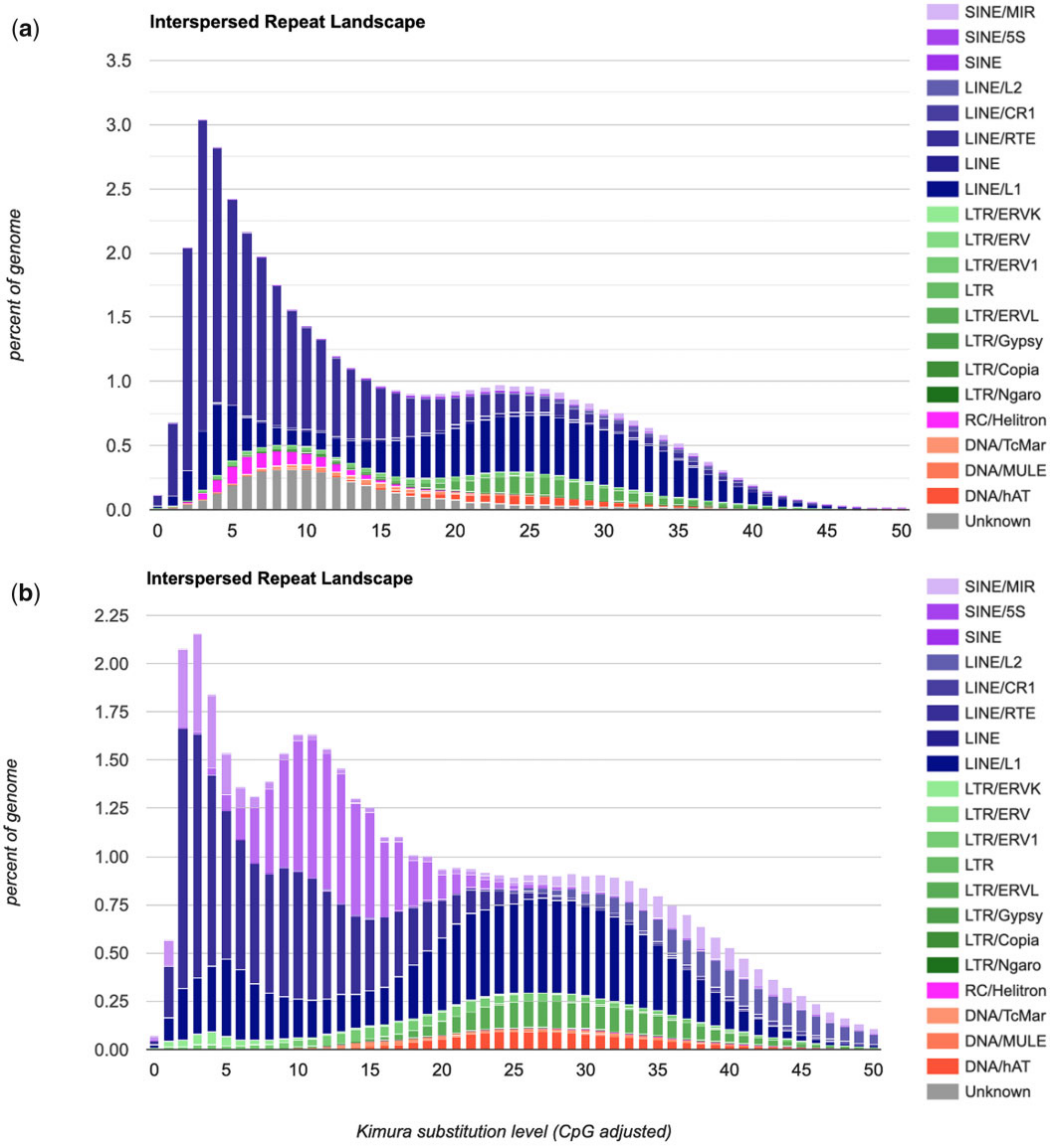
Interspersed repeat landscape of (a) the lowland anoa genome assembled in this study and (b) *Bos taurus*.

Mammalian genomes contain large families of repeats (Goodier and Kazazian 2008), such as long interspersed nuclear elements (LINEs), short interspersed nuclear elements (SINEs), and long-terminal repeats (LTRs). RepeatMasker revealed that 42.12% of the lowland anoa genome is composed of repetitive regions (Table 7), which is comparable to data previously published for genome assemblies of river buffalo and other bovids (Deng *et al.* 2016; Low *et al.* 2019; Mintoo *et al.* 2019; El-Khishin *et al.* 2020). Results also agree with the repetitive content in the cattle genome (Fig. 7b). Both lowland anoa and cattle genomes showed 2 waves of repeat expansion in their repeat landscape (Fig. 7, a and b), suggesting a shared inheritance of such repeats. In the lowland anoa, the LINEs were more abundant, representing 30.04% of the repeats, followed by LTRs representing 3.10% and SINEs representing 1.03% (Table 7).

**Table 7.** Repeat sequence composition of the lowland anoa genome.

Family	Copy number of elements	Length occupied (bp)	% Genome
SINEs	296,064	26,945,915	1.03
LINEs	2,864,468	786,815,034	30.04
LINE1	1,203,360	282,366,346	10.78
LINE2	101,415	13,911,301	0.53
RTE/Bov-B	1,461,651	481,114,012	18.37
LTR elements	362,123	81,208,077	3.10
DNA transposon	255,003	38,433,935	1.47
Small RNA	139,586	14,174,190	0.54
Satellites	269	52,169	0.00
Simple repeats	500,363	20,187,327	0.77
Low complexity	81,685	3,956,146	0.15
Unclassified	611,789	100,086,577	3.82
Total			42.12

## Conclusion

To date, whole-genome sequencing has allowed the identification of variants involved in domestication and genetic improvement for several livestock species (Zimin *et al.* 2009; Canavez *et al.* 2012; Li *et al.* 2020; Rosen *et al.* 2020). However, the lack of wild buffalo genomes hinders further analyses addressing functional and evolutionary aspects of this group, as well as possible conservation efforts. The draft genome assembly of the lowland anoa reported here is expected to contribute to this gap in data availability, as this is the first draft genome assembly for wild Asian buffaloes. Furthermore, we showed that short-read Illumina sequencing data can still provide a cost-effective way of sequencing mammalian genomes to an adequate level of completeness for downstream comparative analyses.

## Data Availability

The raw data and assembly are available on NCBI under BioProject PRJNA849775. The genome assembly of the lowland anoa is available on NCBI under BioSample accession SAMN29133250. The raw data are available on the Sequence Read Archive (SRA) on NCBI under accession SRR21016826.
